# Photoacclimation and Light Thresholds for Cold Temperate Seagrasses

**DOI:** 10.3389/fpls.2022.805065

**Published:** 2022-02-10

**Authors:** Romy Léger-Daigle, Fanny Noisette, Simon Bélanger, Mathieu Cusson, Christian Nozais

**Affiliations:** ^1^Québec-Océan and Institut des Sciences de la Mer de Rimouski, Université du Québec à Rimouski, Rimouski, QC, Canada; ^2^Québec-Océan and Département de Biologie, Chimie et Géographie, Université du Québec à Rimouski, Rimouski, QC, Canada; ^3^Québec-Océan and Département des Sciences Fondamentales, Université du Québec à Chicoutimi, Chicoutimi, QC, Canada

**Keywords:** photophysiology, PAM fluorometry, *Zostera marina*, subarctic, light attenuation and limitation, photosynthesis, eelgrass

## Abstract

Water quality deterioration is expected to worsen the light conditions in shallow coastal waters with increasing human activities. Temperate seagrasses are known to tolerate a highly fluctuating light environment. However, depending on their ability to adjust to some decline in light conditions, decreases in daily light quantity and quality could affect seagrass physiology, productivity, and, eventually, survival if the *Minimum Quantum Requirements* (MQR) are not reached. To better understand if, how, and to what extent photosynthetic adjustments contribute to light acclimation, eelgrass (*Zostera marina* L.) shoots from the cold temperate St. Lawrence marine estuary (Rimouski, QC, Canada) were exposed to seven light intensity treatments (6, 36, 74, 133, 355, 503, and 860 μmol photons m^–2^ s^–1^, 14:10 light:dark photoperiod). Photosynthetic capacity and efficiency were quantified after five and 25 days of light exposure by Pulse Amplitude Modulated (PAM) fluorometry to assess the rapid response of the photosynthetic apparatus and its acclimation potential. Photoacclimation was also studied through physiological responses of leaves and shoots (gross and net primary production, pigment content, and light absorption). Shoots showed proof of photosynthetic adjustments at irradiances below 200 μmol photons m^–2^ s^–1^, which was identified as the threshold between limiting and saturating irradiances. Rapid Light Curves (RLC) and net primary production (NPP) rates revealed sustained maximal photosynthetic rates from the highest light treatments down to 74 μmol photons m^–2^ s^–1^, while a compensation point (NPP = 0) of 13.7 μmol photons m^–2^ s^–1^ was identified. In addition, an important package effect was observed, since an almost three-fold increase in chlorophyll content in the lowest compared to the highest light treatment did not change the leaves’ light absorption. These results shed new light on photosynthetic and physiological processes, triggering light acclimation in cold temperate eelgrass. Our study documents an MQR value for eelgrass in the St. Lawrence estuary, which is highly pertinent in the context of conservation and restoration of eelgrass meadows.

## Introduction

Human-induced environmental stressors contribute to the degradation of light conditions in vegetated coastal ecosystems through changes in water quality. Water quality is especially compromised through increased particle loading in coastal zones from the watershed (Kemp et al., 1983; Hemminga and Duarte, 2000). In addition, excessive anthropogenic nitrogen inputs indirectly limit light penetration in the water column as it stimulates phytoplanktonic and epiphytic algal growth (Kemp et al., 1983; Borum, 1985; Sand-Jensen and Borum, 1991), competing with benthic autotrophs for light (Agusti et al., 1994; Heuvel et al., 2019). The light limitation has been singled out as the primary cause of seagrass loss worldwide (Hauxwell et al., 2001; Short et al., 2011). For example, *Zostera marina* L. (1753; eelgrass), the prevalent seagrass in temperate North Atlantic coastal habitats (Green and Short, 2003), was declining in 2007 at an estimated rate of 1.4% per year (Short et al., 2011). This decline in global spatial cover was attributed to the combined effects of natural environmental pressures (e.g., extreme weather events, ice scouring, and terrestrial runoffs) and anthropogenic disturbances (e.g., land use, sand mining, coastal development, aquatic recreational, and commercial activities) (Green and Short, 2003; Hauxwell et al., 2003; Unsworth et al., 2018) through deterioration of light conditions in coastal waters. Specifically, changes in light intensity, spectral composition, or regime have been shown to strongly impact eelgrass distribution, growth, and survival, and ultimately alter coastal habitats and communities (Dennison, 1987; Zimmerman et al., 1991; Nielsen et al., 2002; Ralph et al., 2007).

Alteration of eelgrass meadow dynamics can profoundly disturb shallow coastal ecosystems because of their critical role in these habitats. Eelgrass meadows provide many ecosystem services and fulfill major ecological roles for coastal communities associated with the complex habitat structure they provide and its associated fauna (Duffy, 2006). Because of its significant ecological role, *Z. marina* was recognized as an Ecologically Significant Species in Canada in 2009 (Cooper et al., 2009). A decline in eelgrass abundance could damper their water purification role through particle depositions and nutrient uptake (Nelson and Waaland, 1997; Hemminga and Duarte, 2000) and contribute even more to the degradation of light conditions in meadows (Maxwell et al., 2017). Eelgrass biological responses to changing light conditions deserve attention, especially in Western North Atlantic coastal waters, where underwater light conditions are altered by sustained human activities (Waycott et al., 2009). This is especially the case in boreal and subarctic environments, where strong seasonality and extreme weather events can cause light attenuation in the water column over periods from a few days to several weeks through, for instance, ice cover, freshet, or terrestrial runoffs, inducing browning of the coastal waters (Murphy et al., 2021).

Changes in light intensity can alter subcellular processes and induce a response to adjust and optimize photosynthesis. As described by Falkowski and Raven (2007), incident light influences the electron transport in the thylakoid membranes (electron transport chain, ETC) downstream of photosystem II (PSII), in which photons are captured by accessory pigments and funneled toward the chl*a* of the reaction center. There, electrons are retrieved from H_2_O molecules to feed the ETC. Electron transport supplies the Calvin cycle with NAPDH and ATP. The most common ways to monitor photosynthesis are through the electron transport rate (ETR) in the ETC and CO_2_/O_2_ fluxes. Manipulation of PSII by Pulse Amplitude Modulated (PAM) fluorometry provides insights into the functioning of the photosynthetic apparatus. This technique reveals the relative importance of the different pathways competing for photon energy: photochemistry, fluorescence, and heat dissipation, complementing the information gathered on primary productivity (Schreiber, 2004).

Following a change in light intensity, biological responses to optimize photosynthesis can occur on different biological and timescales (McMahon et al., 2013; Bertelli and Unsworth, 2018). For example, rapid adjustments to a new constant irradiance take place in a matter of days through subcellular photosynthetic changes (Lambers et al., 2008). On the other hand, photoacclimation, i.e., photosynthetic, physiological, and morphological adjustments to light conditions may take weeks to months, and occur from subcellular to plant scale (McMahon et al., 2013; Schubert et al., 2018). At shoot scale, an increase in leaf surface or photosynthetic biomass, often approximated by an increase of the above or below-ground biomass ratio, helps maintain carbon balance by decreasing the proportion of non-photosynthetic tissues relative to photosynthetic ones (Olesen and Sand-Jensen, 1993). In addition, higher pigment content can counteract low light levels by increasing leaf absorptance (Beer et al., 2014), i.e., the fraction of incident photons harvested by leaf tissues (Kirk, 1994; Zimmerman, 2003). Furthermore, the photosynthetic apparatus responds to low light conditions by optimizing photon use at the subcellular level, thus enhancing photosynthetic efficiency (Bertelli and Unsworth, 2018). However, despite a more efficient photon use, insufficient photon availability leads to a decreased electron transport rate and, consequently, to a lower photosynthetic capacity. Photoacclimation is achieved when the plant has reached a new steady state, reflecting optimization of photosynthesis under its new light environment (Lambers et al., 2008; Bertelli and Unsworth, 2018). At some point, the photophysiological adjustments can no longer compensate for the too few incident photons, and shoot mortality can occur if carbon balance cannot be maintained and metabolic costs exceed carbon fixation by photosynthesis (Longstaff and Dennison, 1999; Ralph et al., 2007; Bertelli and Unsworth, 2018).

There have been attempts to estimate the *Minimum Light Requirements* (MLR) needed for growth and survival for *Z. marina*. This MLR is expressed as a percentage of surface irradiance and traditionally determined by the light intensity measured at the maximum depth limit of a seagrass species or population (Dennison et al., 1993). However, the MLR calculated for seagrasses by Duarte (1991) (i.e., 11% of surface irradiance) is not well suited for cold temperate intertidal ecosystems which experience less daylight than tropical species (Lee et al., 2007; Bulmer et al., 2016; Eriander, 2017). Light requirements can also be regarded as the light intensity under which the shoot respiratory demands amount photosynthesis (Ralph et al., 2007), referred to as *Minimum Quantum Requirements* for growth (MQR) and expressed as photosynthetically active radiation (PAR) intensity. Although rarely encountered in literature (Ralph et al., 2007), this latter proxy is more appropriate when studying photoacclimation in a context of conservation since it provides an absolute minimum light intensity to which seagrasses can acclimate and survive. Therefore, it becomes relevant to reassess these light requirements when studying specific species or even populations, especially for management and conservation purposes (Collier et al., 2012; Bertelli and Unsworth, 2018).

This study aims to characterize the photoacclimation responses of *Z. marina* in controlled conditions along a natural gradient of PAR intensity experienced by an intertidal eelgrass population from the cold temperate St. Lawrence Estuary (Quebec, Canada, ca. 48.5°N). Rapid adjustments to changes in irradiance after five days were quantified by examining tissue-scale photosynthetic responses (i.e., photosynthetic apparatus efficiency and capacity). Photoacclimation was also assessed by examining the evolution of the photosynthetic and physiological adjustments after 25 days of light exposure *via* measurements of photosynthetic apparatus efficiency and capacity, pigment content, and shoot-scale primary production. Based on the observations of Bertelli and Unsworth (2018), shoots metabolism should have reached a new stable state by that time. Compared to physiological responses, the photosynthetic apparatus should respond first, after only a few days of light exposure (Collier et al., 2012; Bertelli and Unsworth, 2018). These rapid adjustments are expected to occur with light decrease until PAR intensity becomes too low to support photosynthetic activity and maintain carbon balance. We hypothesized that photoacclimation would occur as soon as PAR becomes limiting to optimize photon absorption and electron transport, thus maintaining photosynthetic rates. This should be achieved through increased chlorophyll concentration and absorptance, increased photon use, and lower saturating light intensity.

## Materials and Methods

### Sample Collection

Whole eelgrass shoots were collected on the intertidal eelgrass meadow in East Rimouski, Quebec, Canada (48°27′42.24″N 68°31′25.92″O) on July 8, 2020, and placed in a cooler with seawater for transport to the Pointe-au-Père research station located a few kilometers away. The next day, shoots with their root system and surrounding sediments were transplanted into individual plastic cores (5 cm deep, 2.5 cm diameter). Transplanted shoots were approximately 20 cm in height, had intact roots, and three rhizome internodes. Prior to the experiment, shoots were placed in experimental tanks four days for acclimation, with a 14:10 photoperiod (light:dark, h) and under 860 μmol photon m^–2^ s^–1^, which corresponds to the mean light intensity measured over a tide cycle during daytime in the same meadow in summer 2020 (Léger-Daigle, unpublished results).

### Experimental Design and System

Seven PAR treatments (6, 36, 74, 133, 355, 503, and 860 μmol m^–2^ s^–1^) were used to test for eelgrass light adjustment and acclimation responses. This range of light intensities was established to achieve a high resolution of the photoacclimation response in the lower irradiances. Most of the light intensities were chosen for their ecological significance. For instance, 36 and 133 μmol photons m^–2^ s^–1^ are close to the light compensation point for growth and the maximum specific growth rate of *Z. marina*, respectively (Olesen and Sand-Jensen, 1993). Furthermore, the 6 and 860 μmol photons m^–2^ s^–1^ treatments correspond to the mean PAR intensity measured, respectively, under the seasonal sea ice cover in winter (Horner and Schrader, 1982) and during daytime in summer in Rimouski. The latter light treatment, therefore, acts as a control treatment. The other three light intensities (i.e., 74, 355, and 503 μmol photons m^–2^ s^–1^) were selected to achieve exponential increments throughout the studied range.

**FIGURE 1:**
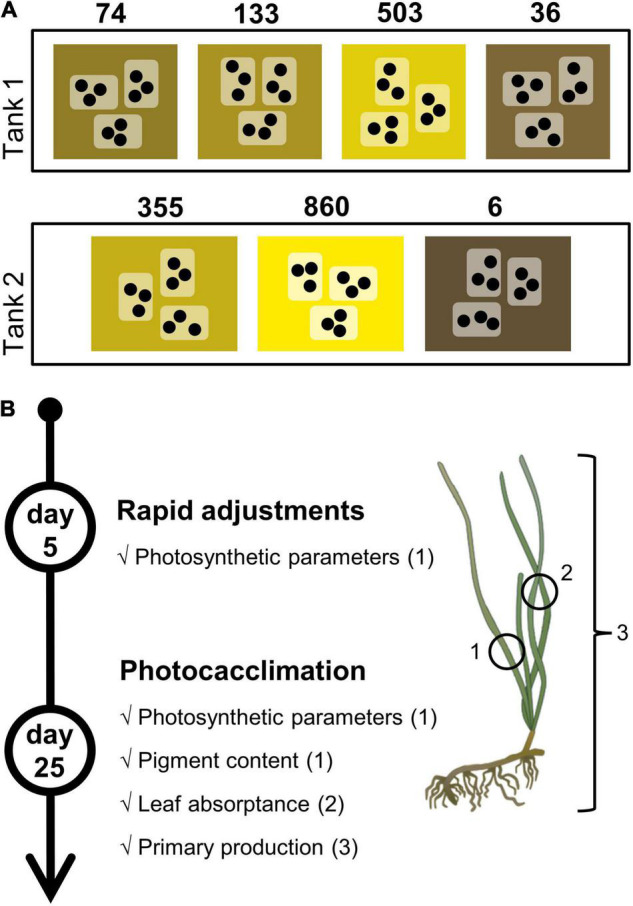
Schematic representation of the experimental system and sampling design. **(A)** Experimental flowthrough system with the two tanks in which were randomly assigned the seven light intensity treatments (numbers in μmol photons m^–2^ s^–1^), to which nine shoots (●: sediment core with a single shoot) were exposed while distributed in three transparent plastic containers. **(B)** Sampling timeline for rapid adjustments and photoacclimation assessment. Numbers in parenthesis refer to the part of the shoot used for measurements. 1: second leaf, 5 cm above the sheath; 2: green mature leaf fragment; 3: whole shoot. For every variable, *n* = 9 shoots per treatment, except for primary production where *n* = 6 shoots per treatment.

The experiment was carried out in a flow-through system in two separate tanks, in which the PAR treatments were randomly assigned (Figure 1). For each PAR treatment, nine shoots were randomly and evenly distributed in three transparent independent containers (three individual plastic cores per container). The shoots served as units of replication, although shoots from the same containers were considered pseudo-replicates and accounted for in the statistical treatment. The containers were continuously and directly supplied with sand-filtered seawater pumped a few kilometers offshore of the research station. Water temperature remained constant at 11 ± 0.01°C. Lighting was ensured by LED growth lights mimicking the sunlight emission spectrum (model GHBH-640W–120V, RayonLed, Montreal, CA, United States). Light intensity was attenuated with gray filters (LEE Filters, Burbank, CA, United States) to reach the targeted PAR, without changing spectral quality. Filters were suspended above the three containers of each treatment. The natural daylight hours of that time of year, a 14:10 photoperiod (light:dark, h), were recreated by an autonomous timer.

Eelgrass shoots were kept in the containers for 25 days, during which epiphytes were manually cleaned off the leaves twice a week. Rapid photosynthetic adjustments were assessed after five days of exposure for each light treatment. Photosynthetic responses were measured again at the end of the experiment (on day 25) to compare these responses to those of day 5 (rapid adjustments) and assess photoacclimation (Figure 1). Physiological responses were also measured on day 25 to appreciate acclimation responses to the different light treatments. Finally, leaf surface (cm^2^) was measured with ImageJ software (Rasband, 2019).

### Photosynthetic Measurements

The photophysiological responses of eelgrass were determined by non-invasive PAM fluorometry with a Diving PAM-II (Heinz Walz GmbH, Effeltrich, Germany). Rapid Light Curves (RLCs) were used to assess photosynthesis (White and Critchley, 1999; Ralph and Gademann, 2005). Particularly, fluorescence is measured through a range of PAR intensity and allows calculation of quantum yields (maximum: Fv/Fm; and effective: YII), ETR, and quenching coefficients (photochemical: qP; and non-photochemical: NPQ) for each actinic light step. RLCs usually exhibit three regions from which photosynthetic parameters can be estimated: (i) in the light-limited region of the RLC, the initial slope of the ETR-PAR relationship (alpha, α) is used as a proxy for photosynthetic efficiency (Schreiber, 2004). (ii) As PAR intensity increases, the onset of light saturation (E_k_) is reached, the ETC saturates and the ETR reaches a plateau (ETR_max_) which serves as a proxy for photosynthetic capacity (Schreiber, 2004). Mathematically, E_k_ corresponds to the intersection of alpha and ETR_max_. Physiologically, E_k_ is the light intensity where neither photochemical reactions (qP) nor heat dissipation (NPQ) dominates fluorescence quenching (Henley, 1993). (iii) In the high end of the PAR range of the RLC, a drop of the ETR can occur, indicating photoinhibition (Henley, 1993).

Rapid Light Curves (RLCs) were carried out on each shoot, on the second leaf, approximately 5 cm above the top of the sheath (Beer et al., 2001), after five and 25 days of light exposure, and around midday every time. The RLCs consisted of 10 actinic light steps (38, 68, 98, 137, 190, 288, 432, 637, 954, and 1246 μmol photons m^–2^ s^–1^) lasting 10 s each. Leaves were shaded with the leaf clip no more than 10 s before the start of the RLC (Ralph and Gademann, 2005). The PAM calculates the ETR using the equation of Beer et al. (2001):


(1) ETR=YII×PAR×AF×0.5

where YII is the effective quantum yield of photosystem II, PAR corresponds to the actinic light intensity generated by the PAM, AF refers to the absorption factor, and 0.5 refers to the even distribution of photons between PSII and PSI (Beer et al., 2001). The YII is automatically calculated based on fluorescence ratios, according to Genty et al. (1989). The AF was set at 0.44, corresponding to the mean fraction of absorbed light for eelgrass (Beer et al., 1998). This AF value of 0.44 was established for populations from higher latitudes (66°N) and is, therefore, not entirely appropriate for our study. It was, however, used in this study to compare results between the beginning and the end of the experiment, and with other studies. This default AF was later replaced by other absorption factors, which were determined spectrophotometrically (see section “Light absorption”) for more accurate calculations of ETRs.

The ETR values were then fitted against the PAR steps to the double exponential decay function described in Platt et al. (1980) to extract the photosynthetic parameters alpha, E_k_, and ETR_max_. This was performed with the software R (R Core Team, version 4.1.1) using the *fitPGH* function and a Port regression algorithm (*fitmethod*) in the Phytotools package (Silsbe and Malkin, 2015). RLCs with no saturation of the electron transport, even at the highest actinic light, were omitted from the analysis since they reflected underlying technical problems.

### Physiological Measurements

#### Pigment Composition

At the end of the experiment, the second leaf of every single shoot was collected and stored at −80°C for pigment analysis. Leaves were ground using a mortar and pestle in 100% acetone on ice and under green light. Photosynthetic and accessory pigments were extracted in 10 ml acetone for 20 h. Upon extraction, chl*a*, chl*b*, and total carotenoids were quantified spectrophotometrically by measuring absorbance at 470, 645, and 662 nm using a Genesys 10UV Scanning (Thermo Electro Corporation, Madison, WI, United States). Pigment concentrations were calculated using Lichtenthaler (1987) equations and standardized to leaf fresh weight (FW).

#### Light Absorption

Since ETR is estimated based on the absorbed PAR, the method for quantifying the fraction of absorbed light can significantly influence the measured photosynthetic rates (Runcie and Durako, 2004). According to Ralph et al. (2007), leaf absorptance should be corrected for light absorption by non-photosynthetic components of photosynthetic tissues. However, the Absorption Factor (AF) used for ETR calculations (Equation 1) is often estimated in a way that makes it impossible to differentiate non-photosynthetic from photosynthetic light absorption (Durako, 2007). Furthermore, the relationship between pigment content and light absorption makes it inadequate to use a single absorption factor for photoacclimation studies (Manassa et al., 2017).

Light absorption was determined using a Lambda850 spectrophotometer (PerkinElmer, Waltham, MA, United States) equipped with a 150 mm integrating sphere. Absorptance measurements were performed on samples collected at the end of the experiment and on any green and mature leaf remaining after pigment analysis. Leaf fragments were suspended at the center of the integrating sphere with a clip-style sample holder (Labsphere Inc., North Sutton, NH, United States) (Moss and Loomis, 1952; Boss et al., 2018). The reflectance ports of the sphere were closed with a white Spectralon reflectance standard, and the beam was angled by 85°. This configuration represents an optimal geometry of absorbance measurement by ensuring the detection of nearly all photons scattered by the leaf. To our knowledge, this technique has never been used for *Zostera marina* leaf absorptance. The spectral absorbance (*D*λ) was converted into leaf spectral absorptance (*A*λ) as


(2) $$A\,\lambda =\left(1-10^{-D\,\lambda }\right)$$

Leaf AF was calculated as the spectral average of *A*λ between 400 and 700 nm. We distinguished the total absorption factor from the absorption factor due to the photosynthetic components of the leaf. Therefore, AF_total_ represents the fraction of absorbed light by the leaf’s photosynthetic and non-photosynthetic components. The measured absorptance was corrected for non-photosynthetic light absorption by subtracting the absorptance in the near infrared (at 750 nm), assumed to be non-photosynthetic (Rühle and Wild, 1979; Cummings and Zimmerman, 2003; Durako, 2007). Photosynthetic absorptance (*A*_*P*_λ) was, thus, obtained with the following correction:


(3) $$A_{P}\,\lambda =\left(A\,\lambda -A_{750}\right)$$

where *A*_*750*_ is the total leaf absorptance at 750 nm. Leaf photosynthetic absorptance (AF_photo_) was calculated as the spectral average of *A*_*P*_λ between 400 and 700 nm. The estimated AF_total_ and AF_photo_ were used for the correction of photosynthetic rates (ETR_max_) *a posteriori*.

#### Primary Production and Respiration

Net primary production (NPP) and respiration (R) were assessed at the end of the experiment by measuring the variation of O_2_ concentration during light and dark incubations, respectively, using a non-invasive oxygen meter Fibox4 (PreSens, Regensburg, Germany) (Noisette et al., 2013). Each shoot was gently cleaned of epiphytes and sediments and individually incubated in 0.2 μm filtered seawater in a 300 ml sealed glass bottle. Each incubation lasted 3 h, during which four measurements were made 20 min apart in the dark and then under the respective light treatment. Bottles were gently shaken every 10 min. Incubations were run in a water bath to keep the temperature close to 11°C. Net and gross primary production (GPP) and respiration rates were calculated using the following equations:


(4) $$N\,P\,P=\frac{\left(\alpha \,l\,i\,g\,h\,t*v\,o\,l\right)}{p\,h\,o\,t\,o\,s\,y\,n\,t\,h\,e\,t\,i\,c\,l\,e\,a\,f\,s\,u\,r\,f\,a\,c\,e}$$

(5) $$G\,P\,P=\frac{\left(\alpha \,l\,i\,g\,h\,t*v\,o\,l\right)-\left(\alpha \,d\,a\,r\,k*v\,o\,l\right)}{p\,h\,o\,t\,o\,s\,y\,n\,t\,h\,e\,t\,i\,c\,l\,e\,a\,f\,s\,u\,r\,f\,a\,c\,e}$$

(6) $$R=\frac{\alpha \,d\,a\,r\,k*v\,o\,l}{t\,o\,t\,a\,l\,l\,e\,a\,f\,s\,u\,r\,f\,a\,c\,e}$$

where α*light* and α*dark* are the slopes of the oxygen concentration variation along time (μmol O_2_ L^–1^ h^–1^), respectively for the light and dark incubations, and *vol* is the volume of the glass bottles (L). GPP and NPP were standardized to photosynthetic leaf surface (only the green parts of the leaves in cm^2^), whereas respiration was standardized to the total leaf surface. Leaf surfaces represent only one side of the leaves. Rates are expressed as μmol O_2_ cm^–2^ h^–1^.

### Statistical Analyses

Relationships between photosynthetic and physiological parameters against light treatment were modeled by fitting hierarchical generalized additive models (HGAM) (Pedersen et al., 2019). The maximum of basis functions was set to *k* = *7*, since light intensity, the principal predictor, had seven levels (even though it was treated as a continuous variable). The identity of the containers in which shoots were kept during the experiment was included as a random factor to account for any undesired added variance among containers.

For photosynthetic parameters analysis, HGAMs were structured with date-specific smoothers to account for the additional temporal aspect of the data (day 5 and day 25). This allowed appreciating the evolution of the functional response between the beginning (rapid adjustment responses) and the end (acclimation responses) of the experiment. Shoot id was also included in the model as a random variable. HGAM for analysis of the corrected ETR_max_ was structured in the same way, only with the method for absorptance estimation (default AF of 0.44, spectrophotometrically measured AF_total_ and AF_Photo_) as a grouping factor.

Gross primary production measured at the shoot scale was fitted to an HGAM model rather than a classic photosynthesis-irradiance (PI) curve. A PI curve usually follows the photosynthetic rate of an individual throughout a range of increasing light intensities (Falkowski and Raven, 2007). Here, the curve is shaped by multiple individuals, all of which are acclimated to their respective light environments (*x*-axis). Therefore, the physiological mechanisms behind the observed response are not the same as with a classic PI curve.

Graphical analysis of the models sometimes suggested thresholds. In these cases, T-tests were carried out to confirm the presence of such a threshold in the response of a variable among light treatments. This was done for both alpha and E_k_ on day 5, between 133 and 355 μmol photons m^–2^ s^–1^. Data were tested for normality and homoscedasticity with the Shapiro and Fligner tests, respectively, and using light treatment as a factor. Statistical analyses were carried out with R (R Core Team, version 4.1.1).

## Results

### Rapid Photosynthetic Adjustments

#### Photosynthetic Efficiency and Capacity

**FIGURE 2:**
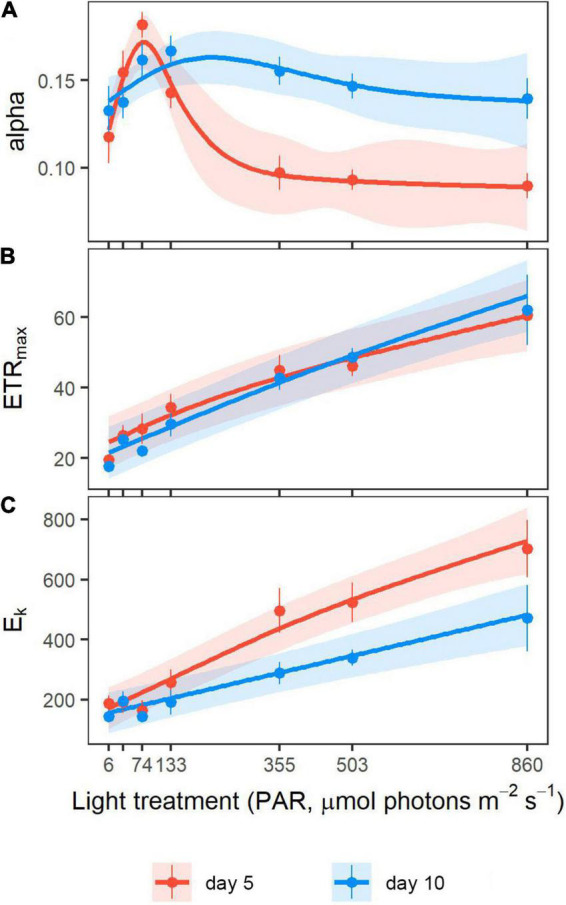
Photosynthetic parameters derived from Rapid Light Curves (RLCs) after 5 (red) and 25 (blue) days of light exposure. Dotted values are mean ± SE (*n* = 5–9 shoots) for each light treatment. Lines correspond to the values fitted by hierarchical generalized additive models (HGAM) with a 95% CI. **(A)** Alpha (initial slope of the RLC), **(B)** maximum electron transport rate (ETR_max_, μmol electrons m^–2^ s^–1^), and **(C)** onset of light saturation (E_k_, μmol photons m^–2^ s^–1^).

Five days after the beginning of the light exposure, photosynthetic efficiency, estimated with alpha, varied significantly with irradiance exposure (*p* < 0.001, Table 1) and followed a non-linear trend. Eelgrass shoots from the 74 μmol photons m^–2^ s^–1^ treatment showed the most efficient electron transport at a low light intensity, as indicated by the peak of alpha at 0.173 (Figure 2A). Above and below this irradiance, alpha decreased strongly. The ETR_max_ increased linearly with the increase of irradiance exposure (*p* < 0.001, Figure 2B), ranging from 24.6 to 62.7 μmol electrons m^–2^ s^–1^ at 6 and 860 μmol photons m^–2^ s^–1^, respectively. On day 5, the onset of light saturation (E_k_) increased significantly with light treatment (*p* < 0.001; Figure 2C).

**TABLE 1:** Output of the hierarchical generalized additive models (HGAM) analyses.

		*F*	*p-*value	*R*^2^ (adj.)	Dev. expl.	*n*
Alpha	date	3.807	0.000239*	0.433	47.9%	113
	s(PAR) on day5	12.775	< 0.001*			
	s(PAR) on day25	1.381	0.187			
	s(container)	0.212	0.300			
	s(id)	0.000	0.735			
ETR_max_	date	–1.037	0.302	0.72	78%	113
	s(PAR) on day5	38.725	< 0.001*			
	s(PAR) on day25	72.502	< 0.001*			
	s(container)	0.001	0.4356			
	s(id)	0.494	0.0394*			
E_k_	date	–4.135	7.29e−05*	0.62	65.3%	113
	s(PAR) on day5	60.507	< 0.001*			
	s(PAR) on day25	33.609	< 0.001*			
	s(container)	0.001	0.456			
	s(id)	0.112	0.307			
corrected ETR_max_	0.44-AF	4.094	6.88e−05*	0.781	79%	159
	0.44-AP	–12.987	< 0.001*			
	AF-AP	–17.081	< 0.001*			
	s(PAR) with 0.44	42.732	< 0.001*			
	s(PAR) with AF	116.152	< 0.001*			
	s(PAR) with AP	14.177	0.000237*			
	s(container)	0.058	0.349388			
Chl*a*	s(PAR)	31.53	< 0.001*	0.551	56.5%	58
	s(container)	0.000	0.593			
Chl*b*	s(PAR)	19.73	2.24e−07*	0.45	47%	58
	s(container)	0.000	0.711			
Carotenoids	s(PAR)	8.759	0.00247*	0.153	17%	58
	s(container)	0.000	0.90185			
AF_total_	s(PAR)	3.535	0.0324*	0.0868	11.2%	83
	s(container)	0.000	0.4054			
AF_photo_	s(PAR)	1.139	0.413	0.0111	2.97%	83
	s(container)	0.000	0.996			
GPP	s(PAR)	13.189	1.8e−06*	0.568	61.7%	41
	s(container)	1.315	0.112			
NPP	s(PAR)	91.013	< 0.001*	0.863	87.4%	82
	s(container)	3.504	0.0142*			

The table shows the F statistics and p-values for each predictor, and adjusted R-squared (R^2^ adj.), deviance explained and sample size (n) for each model. Smoothed variables are identified with s(). Statistical significance is identified with an asterisk (*).

As shown by the patterns depicted by the models for the three photosynthetic parameters, functional responses reached a threshold of around 200 μmol photons m^–2^ s^–1^ after five days of experimenting. Alpha and E_k_ were significantly different under 133 μmol photons m^–2^ s^–1^ compared to 355 μmol photons m^–2^ s^–1^ (T-test, *p* = 0.005 and 0.023, respectively). Both parameters did not vary significantly above 355 μmol photons m^–2^ s^–1^. Furthermore, E_k_ did not change with light treatment beneath 133 μmol photons m^–2^ s^–1^, with mean values close to 200 μmol photons m^–2^ s^–1^.

### Photoacclimation

#### Photosynthetic Apparatus Comparison Between Day 5 and Day 25

The relationship between alpha and light treatment changed significantly between day 5 and day 25 (*p* < 0.001, Table 1 and Figure 2A), leading at the end to a consistent alpha among all the light treatments (*p* = 0.187, HGAM). As of 355 μmol photons m^–2^ s^–1^ and above, alpha significantly increased between day 5 and day 25 based on the non-overlapping confidence intervals (Crawley, 2013). The increase in ETR_max_ with light treatments was similar on day 5 and day 25 (*p* = 0.302), as supported by the overlapping confidence intervals. Conversely, E_k_ increased differently with light treatment on day 5 and day 25 (*p* < 0.001, Table 1), showing a greater slope at day 5 compared to day 25. The CIs for the two dates cease to overlap as of 355 μmol photons m^–2^ s^–1^ and beyond.

#### Pigments and Light Absorption

**FIGURE 3:**
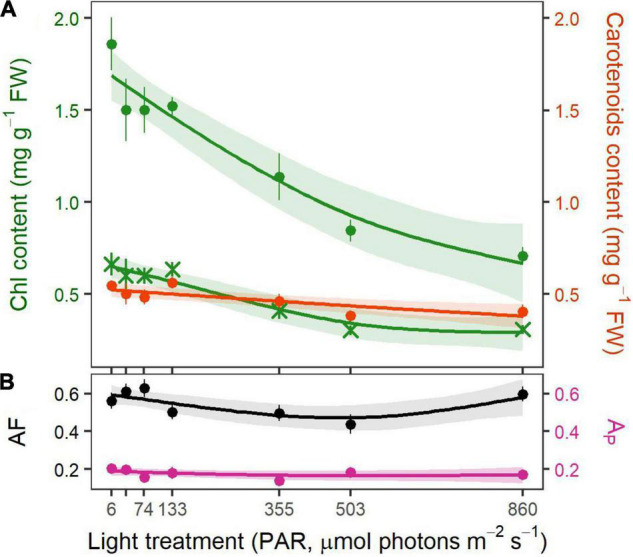
Pigment content and absorptance of eelgrass leaves for each light treatment at the end of the experiment. **(A)** Chl*a* (green circles), chl*b* (green x), and total carotenoids (orange) contents (mg pigment g^–1^ FW). **(B)** Total absorption factor (AF_total_, black) and photosynthetic absorption factor (AF_photo_, purple). Dotted values are mean ± SE (*n* = 8–9 shoots). Lines represent the fitted values from HGAM models with a 95% CI.

After 25 days of light exposure, chlorophyll contents in the eelgrass leaves decreased with increasing irradiance (*p* < 0.001 for both chl*a* and chl*b*, Table 1). Chl*a* and chl*b* contents were over two times higher in the four lower light treatments (133 μmol photons m^–2^ s^–1^ and beneath) than at 860 μmol photons m^–2^ s^–1^ (Figure 3A). Total carotenoids followed a similar trend, although the relationship was less pronounced (*p* = 0.002, Table 1).

The light harvesting efficiency (AF_total_) of eelgrass leaves was minimal in the mid-range irradiances (*p* = 0.032, Table 1 and Figure 3B). AF_total_ ranged from 0.47 in the 503 μmol photons m^–2^ s^–1^ treatment to 0.59 and 0.58 under 6 and 860 μmol photons m^–2^ s^–1^, respectively. Photosynthetic absorptance (AF_photo_, *p* = 0.413, Table 1) did not change with light treatment. By the end of the experiment, eelgrass shoots captured on average 55% (AF_total_ = 0.55 ± 0.02 SE) of incident light while only 18% (AF_photo_ = 0.18 ± 0.01 SE) of incident photons were trapped by the photosynthetic apparatus.

#### Correcting Electron Transport Rates for Photosynthetic Light Absorption

**FIGURE 4:**
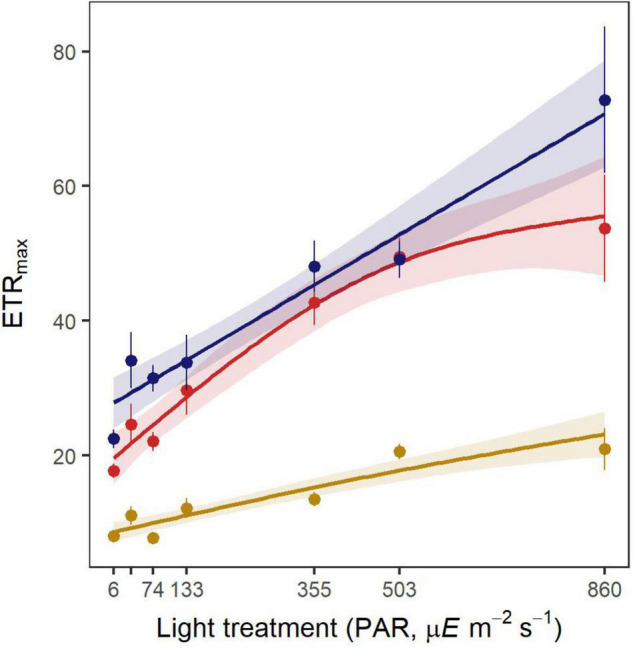
Maximum electron transport rates (ETR_max_, μmol electrons m^–2^ s^–1^) for each light treatment at the end of the experiment. Dotted values are mean ± SE (*n* = 5–9 shoots) uncorrected ETR_max_ (red, with default AF of 0.44) and rates corrected with the measured AF_total_ and AF_photo_ (blue and yellow, respectively). Lines represent the fitted values from HGAM models with a 95% CI.

Correction of the electron transport rates, by replacing the default AF value of 0.44 in the ETR equation with the measured AF_total_ (refer to section “Light Absorption”), significantly affected the relationship between ETR_max_ and light treatment by increasing its intercept rather than the overall trend (*p* < 0.001, Figure 4). Further correction of the photosynthetic rates with the AF_photo_ led to a stronger change of the relationship (*p* < 0.001), yielding to ETR_max_ values 67% lower than the rates calculated with the default AF (Figure 4). ETR_max_ increased significantly with increasing irradiance, regardless of the method for absorptance estimation (HGAM model, Table 1, *p* < 0.001 with default AF, AF_total_, and AF_photo_).

#### Shoot-Scale Primary Production

**FIGURE 5:**
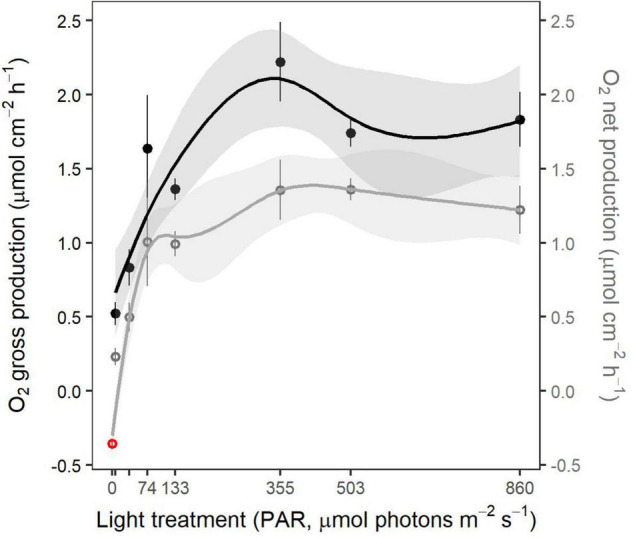
Net primary production (NPP) and gross primary production (GPP) standardized by photosynthetic leaf surface (μmol O_2_ h^–1^ cm^–2^) of whole eelgrass shoots from each light treatment at the end of the experiment. Dotted values are mean ± SE (*n* = 5–6 shoots). NPP at 0 μmol photons m^–2^ s^–1^ (red circle) is the mean respiration rate measured throughout the range of light treatments (*n* = 41). Lines represent the fitted values from HGAM models with a 95% CI.

Gross primary production rates increased from 0.56 μmol O_2_ cm^–2^ h^–1^ in the lowest light treatment up to a peak of 2.04 μmol O_2_ cm^–2^ h^–1^ at 355 μmol photons m^–2^ s^–1^ (Figure 5). NPP rates increased from 0.29 to 1.0 μmol O_2_ cm^–2^ h^–1^, from the 6 to the 74 μmol photons m^–2^ s^–1^ treatments. Above that irradiance level, NPP reached a plateau (Figure 5). Dark respiration (R) rates in the 355 and 860 μmol photons m^–2^ s^–1^ treatments averaged −0.55 ± 0.08 SE and −0.53 ± 0.03 SE μmol O_2_ cm^–2^ h^–1^, respectively, whereas the other light treatments yielded an overall mean respiration rate of −0.28 ± 0.02 SE μmol O_2_ cm^–2^ h^–1^.

An MQR for our light acclimated eelgrass shoots was estimated from the NPP-irradiance HGAM model considering a mean respiration rate of 0.35 ± 0.02 SE μmol O_2_ cm^–2^ h^–1^ for a light intensity of 0 μmol photons m^–2^ s^–1^. This MQR was estimated to occur at 13.7 μmol photons m^–2^ s^–1^, the irradiance at which photosynthesis (GPP) would equal respiration (NPP = 0).

## Discussion

**FIGURE 6:**
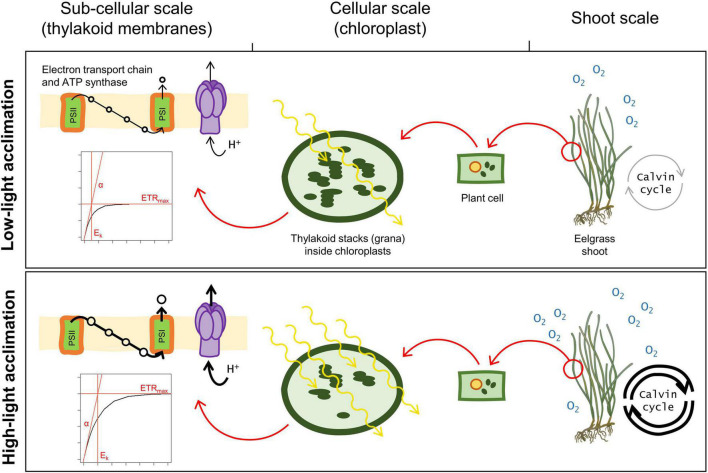
Photoacclimation responses of eelgrass shoots to low and high light, observed after 25 days of light exposure. The limit between low and high light was drawn at 200 μmol photons m^–2^ s^–1^, based on E_k_ measurements. The low-light acclimation response illustrated here only sums up the observations made for the 74 and the 133 μmol photons m^–2^ s^–1^ treatments, which are irradiances for which photoacclimation was sufficient to prevent any shoot decay. The figure shows the highest photosynthetic capacity (ETR_max_) and the onset of light saturation (E_k_) of the high-light acclimated shoots compared to the low-light acclimated ones, suggesting a more efficient electron transport chain. Photosynthetic efficiency (alpha) was similar in both light conditions. Higher pigment contents in low-light acclimated shoots are illustrated by an increased number of thylakoids inside the chloroplasts. Light absorption, represented by the wavy yellow arrows, was constant amongst light conditions. Low-light acclimated shoots had lower primary production (GPP), and presumably a lower Calvin cycle activity than high-light acclimated ones.

This experimental study assessed the capacity of *Z. marina* shoots to adjust and acclimate to light through a broad range of irradiances from 6 to 860 μmol photons m^–2^ s^–1^. Short-term photosynthetic adjustments measured after five days of exposure and photoacclimation processes after 25 days were observed in response to light attenuation. Rapid adjustments of the photosynthetic apparatus after five days revealed a light intensity threshold between 133 and 355 μmol photons m^–2^ s^–1^ at which photosynthetic parameters started to change compared to the higher light treatments. Furthermore, photoacclimation revealed a second threshold around 74 μmol photons m^–2^ s^–1^ at which photoacclimation mechanisms were optimal (Figure 6) and below which photosynthesis and primary production were impeded.

### Rapid Photosynthetic Adjustments

Rapid responses of the photosynthetic apparatus already occurred after five days of exposure to new light conditions, as previously demonstrated by other studies (Collier et al., 2012; Bertelli and Unsworth, 2018). Globally, the photosynthetic capacity (ETR_max_) increased linearly with increasing light treatment, hence leading to the increase of the saturation irradiance (E_k_), as reported in previous studies (Ralph and Gademann, 2005; Ochieng et al., 2010; Bertelli and Unsworth, 2018) and is a direct consequence of the light limitation of the electron transport chain (ETC). The peak of photosynthetic efficiency (alpha) reached under 74 μmol photons m^–2^ s^–1^, and its plateau above 355 μmol photons m^–2^ s^–1^ suggests that electron transport was most efficient at 74 μmol photons m^–2^ s^–1^. From 133 μmol photons m^–2^ s^–1^ and below, E_k_ had stabilized around 200 μmol photons m^–2^ s^–1^, which is higher than the treatment intensity. These changes illustrate the optimization of electron input into the ETC when incident PAR intensity decreases. Above 355 μmol photons m^–2^ s^–1^, alpha stabilized, implying that these light intensities did not necessitate any photosynthetic adjustments from the shoots, probably because they were closer to the natural PAR intensities to which the shoots were acclimated *in situ* at the time of collection (mean irradiance of ca. 860 μmol m^–2^ s^–1^ during daylight hours in July). Photosynthetic adjustments occurred below 355 μmol photons m^–2^ s^–1^, as evidenced by the increased photosynthetic efficiency (alpha). The irradiance of their implementation is, therefore, somewhere between 133 and 355 μmol photons m^–2^ s^–1^.

Despite adjustments of alpha, processes on the acceptor side of PSII caused a limitation of ETR_max_ in low-light treatments. Indeed, a rate-limiting step in the ETC or a slow Rubisco activity associated with low-light conditions can lower the maximum rate of electron transport by slowing down the turnover rate (or reoxidation) of PSII (Sukenik et al., 1987; Han, 2001; Behrenfeld et al., 2004). Our results suggest photosystem turnover was much slower in shoots from the 133 μmol m^–2^ s^–1^ light treatment and beneath than the higher treatments. This is supported by the sharp increase of the fluorescence signal (F) in these lower irradiances (Ralph and Gademann, 2005), as revealed by the fluorescence kinetics obtained during RLCs (Supplementary Figure 1). This increasingly limited capacity of the ETC, accompanied by an increase of alpha, leads to its quicker saturation (E_k_). This dynamic between the three photosynthetic parameters amongst themselves and with light intensity is a characteristic response of the photosynthetic apparatus to highly fluctuating irradiances (Behrenfeld et al., 2004). This potential for such rapid photosynthetic adjustments enables eelgrass to achieve efficient photosynthesis in the highly variable light conditions of the intertidal zone (Anthony et al., 2004; Manassa et al., 2017; Bertelli and Unsworth, 2018).

### Photoacclimation

After 25 days of light exposure, the photosynthetic responses differed partly from those observed after five days. In the three highest light treatments (355, 503, and 860 μmol m^–2^ s^–1^), alpha increased between the beginning and the end of the experiment, leading the shoots to exhibit similar efficiencies regardless of the treatment. However, their ETR_max_ did not change between days 5 and 25. These shoots showed a decrease in their E_k_ since an increase of alpha unaccompanied by a change of ETR_max_ inevitably leads to a decrease of E_k_. In the lower light treatments (133 μmol m^–2^ s^–1^ and beneath), shoots exhibited little to no change of their photosynthetic parameters between the beginning and the end of the experiment. Accordingly, Bertelli and Unsworth (2018) reported a quick (after five days) and then stable photosynthetic response for similar low light intensities (from 3 to 155 μmol m^–2^ s^–1^).

Photosynthetic responses shifted between 133 and 355 μmol photons m^–2^ s^–1^, supporting the observations made on day 5. The change of the photosynthetic parameters with time in the three above-mentioned highest light treatments and the E_k_ plateau around 200 μmol photons m^–2^ s^–1^ suggest that this specific irradiance level draws the line between limiting and non-limiting irradiances. It is indeed the lowest light intensity needed to saturate the ETC, even after acclimation of the shoots. It was used hereafter to distinguish low-light (i.e., limiting or non-saturating) from high-light (i.e., saturating) treatments. Schwarz (2004) reported a similar saturating light intensity for subtropical intertidal and subtidal shoots of *Zostera capricorni*, with E_k_ ranging from 195 to 242 μmol photons m^–2^ s^–1^. Furthermore, I_k_ values (the equivalent of E_k_, only obtained from PI curves instead of RLCs) ranging from 198 to 220 μmol photons m^–2^ s^–1^ were reported by Goodman et al. (1995) for subtropical *Z*. *marina* regardless of their experimental light exposure.

In high-light treatments, as alpha increased between days 5 and 25, plants likely became better acclimated to low PAR intensities. In other words, these shoots developed a more efficient use of photons when light is scarce, but not when it is saturating (i.e., their respective light treatments). Effective quantum yield (YII) at the light treatment intensity did not change either (Supplementary Figure 2) while it increased from day 5 to day 25 in the low end of the PAR range of the RLCs. This enhanced efficiency under low PAR for high-light acclimated shoots is likely a secondary effect of a structural change in the ETC such as the size or redox state of the plastoquinone pool, PSII:PSI ratio, or trans-thylakoid pH variations (Wilson and Huner, 2000; Yang et al., 2017). The E_k_ measured in plants from high-light treatments dropped by more than 100 μmol m^–2^ s^–1^ between day 5 and 25 which resulted in plants being exposed to irradiances higher than their saturating irradiances. In addition, these shoots exhibited important non-photochemical quenching (NPQ, Supplementary Figure 3) due to greater heat dissipation when the ETC is saturated (Falkowski and Raven, 2007). This important heat dissipation in shoots with a constantly supersaturated ETC may have prevented cellular damage related to oxygen build-up and reactive oxygen species (ROS) formation (Badger, 1985; Ralph et al., 2002).

As for low-light (6–133 μmol m^–2^ s^–1^) acclimated shoots, photosynthetic parameters remained the same as on day 5, with high alpha and low ETR_max_ and E_k_ compared to the high-light treatments. E_k_ remained similar after 25 days of low-light exposure, implying that the ETC still would not saturate with less than 200 μmol photons m^–2^ s^–1^, which is well above the irradiances of the low-light treatments. These findings suggest that photoacclimatory mechanisms were already fully set as of day 5, probably underpinned by the rapid regulation of genes involved in photosynthesis a couple of days after exposure to severe light attenuation (Davey et al., 2018). The NPQ kinetics (Supplementary Figure 3) suggests the preservation of photoprotection mechanisms throughout the range of light tested. The NPQ of low-light acclimated shoots saturated more quickly than in the high-light acclimated shoots, indicating efficient dissipation of excess energy as soon as ETC saturates. Similar NPQ plateaus regardless of the light treatment suggest that maximal photoprotective ability was comparable among treatments, even though it was reached at lower light intensities for low-light acclimated shoots. This can be attributed to the naturally important xanthophyll pool found in plants from highly variable light environments (Demmig-Adams et al., 1999) such as intertidal meadows. These observations differ from those reported by Ralph and Gademann (2005), where low-light (50 μmol m^–2^ s^–1^) acclimated eelgrass shoots had a reduced ability for excess energy dissipation compared to high-light (300 μmol m^–2^ s^–1^) acclimated ones for a similar exposure duration.

Overall, pigment content decreased with increasing light intensity, chl*a* and chl*b*, showing greater variations than carotenoids. This relationship between chl*a* and *b* content and light is consistent with previous studies (Cummings and Zimmerman, 2003; Silva et al., 2013; Bertelli and Unsworth, 2018). The subtle change in carotenoid contents with light intensity can be related to the preservation of heat dissipation mechanisms, as mentioned above, and/or to an optimization of light harvesting in low-light environments (Silva et al., 2013; Davey et al., 2018). Changes in pigment contents are often considered as photoacclimatory mechanisms enabling better light absorption (Ralph et al., 2007; Schubert et al., 2018). However, the adaptation of seagrasses to the aquatic life, consisting of concentrating the chloroplasts in the leaf epidermis to optimize inorganic carbon acquisition (Hemminga and Duarte, 2000; Enríquez, 2005), leads to a strong package effect (Cummings and Zimmerman, 2003; Enríquez, 2005; Durako, 2007). This phenomenon is caused by self-shading of overlapping pigments (Cummings and Zimmerman, 2003) and results in a non-linear relationship between pigment content and light absorption (or absorptance), overriding the influence of pigment content on leaf optical properties. The occurrence of a strong package effect was supported in our study by an almost three-fold increase in chl*a* content in low-light treatments without any significant increase in absorptance (Figure 6).

Leaf absorptance was influenced by the strong natural variability of its optical properties and therefore not considered as a relevant proxy of eelgrass photoacclimatory response. For instance, leaf absorptance can vary substantially within and among shoots and with the physiological state of the photosynthetic tissues (Vähätalo et al., 1998; Enríquez, 2005; Durako, 2007). As previously shown, the photosynthetic capacity increased with PAR intensity, regardless of the absorptance coefficient used. Thus, the choice of absorptance coefficient does not affect the observed functional response of photosynthetic tissues to light intensity. However, the use of absorptance coefficients that are not corrected for non-photosynthetic light absorption (default AF and AF_total_) leads to an important overestimation of photosynthetic rates (Runcie and Durako, 2004). ETRs should always be estimated using photosynthetic absorptance (AF_photo_), especially if those rates are to be compared or linked to other quantitative photosynthetic or physiological parameters. Furthermore, from the lack of relationship between pigment content and leaf absorptance, we can infer that a change in pigment content with time (as an acclimation mechanism) did not affect absorptance. Thus, leaf absorptance probably remained the same throughout the experiment, which makes the comparison of photosynthetic parameters between days 5 and 25 valid even though absorptance was only measured at the end.

Net primary production and GPP rates measured at the shoot scale increased with an irradiance of up to 74 and 355 μmol photons m^–2^ s^–1^, respectively. The peak of GPP at 355 μmol photons m^–2^ s^–1^ suggests that this light intensity at which the highest rates of photosynthesis occur is the light optimum for acclimated eelgrass shoots. Beneath this irradiance (or most likely beneath 200 μmol m^–2^ s^–1^), primary production is limited by light availability. On the other hand, the NPP plateau reached 74 μmol photons m^–2^ s^–1^, well beneath the light optimum suggested by GPP, which can be attributed to higher dark respiration rates at 355 μmol photons m^–2^ s^–1^, affecting the overall shape of the HGAM model. Rates of primary production were over two times higher than those reported by Beer et al. (1998) and Dennison and Alberte (1985, 1986) for acclimated shoots and similar light intensities. This discrepancy can be partly explained by the standardization of oxygen fluxes by leaf surface while the whole shoot (below-ground tissues included) was incubated. Standardization by total dry weight would have been more convenient but was precluded by the destructive nature of pigment and absorptance analyses. However, the NPP plateau is close to the light saturation point of 100 μmol photons m^–2^ s^–1^ for *Z. marina*, defined by Dennison and Alberte (1982, 1985), although this value was estimated through PI curves. The plateau of GPP above 355 μmol photons m^–2^ s^–1^ while electron transport keeps increasing may be explained by an increase in photorespiration (Beer et al., 1998) to counteract oxygen build-up and prevent photodamage (Kozaki and Takeba, 1996). The different saturating intensities for NPP and GPP can be explained by the dark respiration rates, which were two times higher in the 355 and 860 μmol photons m^–2^ s^–1^ treatments.

The *Minimum Quantum Requirements* of 13.7 μmol photons m^–2^ s^–1^, derived from predicted values of the HGAM model, was inside the range of values for compensation points (10–25 μmol photons m^–2^ s^–1^) found by Dennison and Alberte (1982, 1985), although these values were, again, obtained from classic PI curves. The MQR of 13.7 μmol photons m^−2^ s^−1^ (0.69 mol m^−2^ d^−1^ according to our experimental setup) is much lower than the average light intensity (4.91 mol m^−2^ d^−1^) at the minimum depth limit of a New Zealand *Z. muelleri* population, as measured by Bulmer et al. (2016) at the minimum depth limit for a New Zealand *Zostera muelleri* population. This difference between the two studies may be related to species-specific responses to light changes (Touchette and Burkholder, 2000). Our lowest light treatment, a light intensity of 6 μmol m^–2^ s^–1^, would be too low to support primary production. Hence, the impaired photosynthetic efficiency (alpha) and the poor photosynthetic capacity (ETR_max_) measured at 6 μmol photons m^–2^ s^–1^ support the hypothesis for deterioration of the photosynthetic apparatus. NPP, however, was positive at this irradiance, although close to zero. These shoots might have survived off their carbohydrate reserves (rhizomes) for the experiment duration (Olesen and Sand-Jensen, 1993; Ralph et al., 2007; Silva et al., 2013). A longer experiment would have confirmed if 6 μmol photons m^–2^ s^–1^ were insufficient to support basic metabolism, in which case shoot mortality would have been observed once starch reserves depleted.

The findings brought up by this study could be helpful in the context of conservation and restoration of cold temperate *Z. marina* meadows. We identified multiple light thresholds with different ecological and physiological significance. For instance, the lowest PAR intensity at which eelgrass exhibited a positive NPP, identified as the MQR, was around 13.7 μmol m^–2^ s^–1^. However, the maximum NPP was reached around 74 μmol m^–2^ s^–1^ through the implementation of photoacclimation mechanisms. *Z. marina* should further thrive under irradiances around 200 μmol m^–2^ s^–1^ since this PAR intensity was considered as saturating and, thus, did not limit photosynthesis and should allow the build-up of carbohydrate reserves. Similar conclusions were drawn by Thom et al. (2008), reporting minimum requirements of 3 mol photons m^–2^ day^–1^ for long-term survival and of 7 mol m^–2^ day^–1^ for light-saturated growth for a northeastern Pacific eelgrass population from similar latitude. These numbers draw near to our light thresholds of 74 μmol m^–2^ s^–1^ (3.7 mol m^–2^ day^–1^) and 200 μmol m^–2^ s^–1^ (10.1 mol m^–2^ day^–1^), respectively. The light thresholds identified in our study are, in our opinion, more accurate than the information usually obtained from classic PI curves. Furthermore, using production rates from acclimated shoots provide insights into the photoacclimatory potential of this species or population, whereas PI curves rather reflect acclimation to one specific light intensity. Therefore, the MQR of 13.7 μmol photons m^–2^ s^–1^ and the saturating irradiance of 200 μmol m^–2^ s^–1^ are more useful in a context of conservation than the usual compensation (I_c)_ and saturation (I_sat_) points derived from PI curves. However, the light intensities used in our study did not mimic natural light regimes, which are governed by photoperiod, tides, and water column light attenuation variability. Thus, the thresholds identified here must be seen as averages instead of integrated light intensities (mol photons m^–2^ day^–1^). An experimental setup with light treatments recreating natural photoperiods would have allowed calculating representative daily PAR intensities. Nonetheless, our results give valuable insights into the photoacclimatory ability of *Z. marina* and highlight key compensatory mechanisms encompassing different biological scales and allowing them to thrive in very fluctuating light environments.

### Ecological Implications and Concluding Remarks

Our study demonstrated the ability of the *Zostera marina* to maintain its photosynthetic rates throughout an extensive range of irradiances through a quick response of its photosynthetic apparatus to changing light intensity. Under experimental conditions, these adjustments were only observed beneath 200 μmol photons m^–2^ s^–1^, which was here identified as the threshold between limiting and saturating irradiances. After five days of light exposure, shoots from light-limited treatments had already implemented photoacclimatory mechanisms through increased photosynthetic efficiency and lower photosynthetic capacity. Shoots exposed to non-limiting irradiances exhibited a slower acclimation. Primary production rates measured after 25 days of light exposure resulted from underlying changes at cellular and subcellular scales. In high-light acclimated shoots, light intensity exceeded what was needed for ETC saturation (E_k_), which likely underpinned photoprotective mechanisms (photorespiration and heat dissipation through NPQ). Once light became limiting (as of 200 μmol m^–2^ s^–1^ and beneath), photoacclimation allowed shoots to maintain photosynthetic rates and carbon balance, as illustrated by the NPP plateau from 74 μmol photons m^–2^ s^–1^ and above. Beneath this light intensity, primary production was not maximal, because limited by light availability but still positive. The apparent optimization of photosynthetic efficiency, regardless of the light treatment, as evidenced by alpha on day 25, supports the ability of eelgrass to acclimate to a wide range of light intensities. Severe light limitation, i.e., when irradiance falls beneath the MQR of 13.7 μmol photons m^–2^ s^–1^, possibly led to a deterioration of the photosynthetic apparatus and consumption of carbohydrate reserves. A reduction of underwater light intensity beneath 13.7 μmol m^–2^ s^–1^ for a prolonged period caused, for instance, by drastic eutrophication, intense sustained human activities (e.g., dredging), or by local sea-level rise could have an impact at meadow-scale through shoot density decline, narrower distribution area, or shoaling of the meadow.

The ability to quickly respond to changing light conditions is critical in cold temperate intertidal ecosystems where underwater light intensity can change considerably and rapidly over a tidal cycle with weather conditions and depending on the seasons (Anthony et al., 2004). Shoots have demonstrated a quick response to sudden light limitation and high tolerance to high intensities. Photoacclimation ability is as important as quick adjustments to changing light in the long-term. Indeed, seagrass habitats are expected to change, especially with regard to underwater light conditions, with climate change and human-induced disturbances (Hauxwell et al., 2003; Short et al., 2011). Seagrasses with a high potential for photoacclimation would cope better with these changes.

## Data Availability Statement

The raw data supporting the conclusions of this article will be made available by the authors, without undue reservation.

## Author Contributions

RL-D, FN, CN, and MC devised the work plan for the experiment. RL-D and FN carried out the experiments. RL-D carried out the sample and statistical analyses, with input from FN, MC, and CN. RL-D wrote the first manuscript, which was then improved with editorial inputs from all authors. All authors have read and agreed to the published version of the manuscript.

## Conflict of Interest

The authors declare that the research was conducted in the absence of any commercial or financial relationships that could be construed as a potential conflict of interest.

## Publisher’s Note

All claims expressed in this article are solely those of the authors and do not necessarily represent those of their affiliated organizations, or those of the publisher, the editors and the reviewers. Any product that may be evaluated in this article, or claim that may be made by its manufacturer, is not guaranteed or endorsed by the publisher.

## Supplementary Material

Supplementary Figure 1 Maximum (Fm’, dashed lines) and steady-state fluorescence signals (F, solid lines) for each light treatment (μmol photons m^–2^ s^–1^) during Rapid Light Curves (RLCs) at the beginning and the end of the experiment (day 5 in red and day 25 in blue). Lines are mean values (*n* = 5–9) with a 95% CI.

Supplementary Figure 2 Effective quantum yield (YII) of photosystem II (PSII) (dashed lines) and electron transport rate (ETR, solid lines) for each light treatment (μmol photons m^–2^ s^–1^) during RLCs at the beginning and the end of the experiment (day 5 in red and day 25 in blue). Lines are mean values (*n* = 5–9) with a 95% CI.

Supplementary Figure 3 Photochemical coefficients (qP, dashed lines) and non-photochemical quenching coefficients (NPQ, solid lines) for each light treatment (μmol photons m^–2^ s^–1^) during RLCs at the beginning and the end of the experiment (day 5 in red and day 25 in blue). Lines are mean values (*n* = 5–9) with a 95% CI.
