# Supplementary figures and images for: Photoacclimation and Light Thresholds for Cold Temperate Seagrasses
Source: Front Plant Sci. 2022 Feb 10;13:805065. doi: 10.3389/fpls.2022.805065 (PMC8866642; doi:10.3389/fpls.2022.805065)

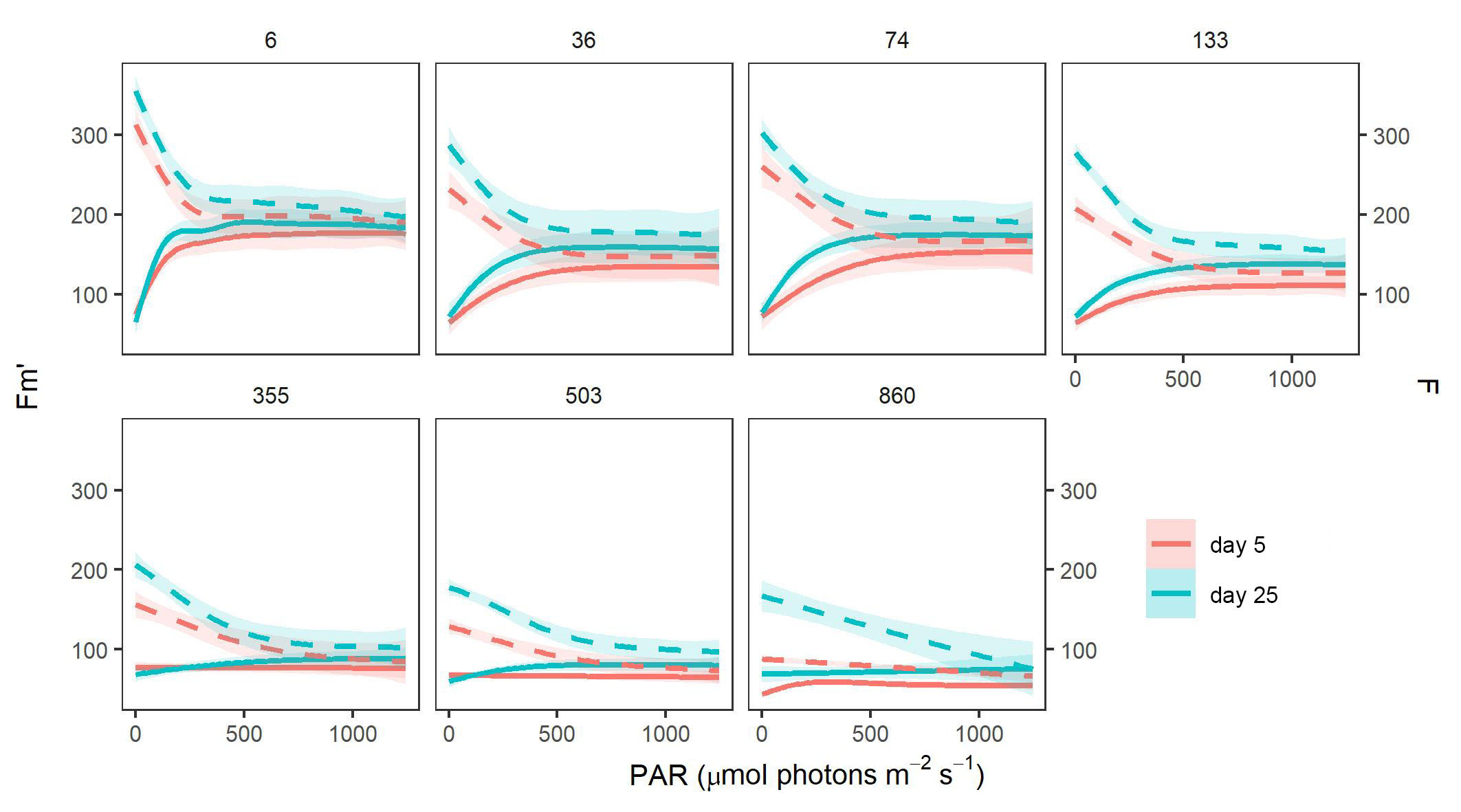

Supplement: Supplementary Figure 1 — Maximum (Fm’, dashed lines) and steady-state fluorescence signals (F, solid lines) for each light treatment (μmol photons m–2 s–1) during Rapid Light Curves (RLCs) at the beginning and the end of the experiment (day 5 in red and day 25 in blue). Lines are mean values (n = 5–9) with a 95% CI. [file Image_1.JPEG]

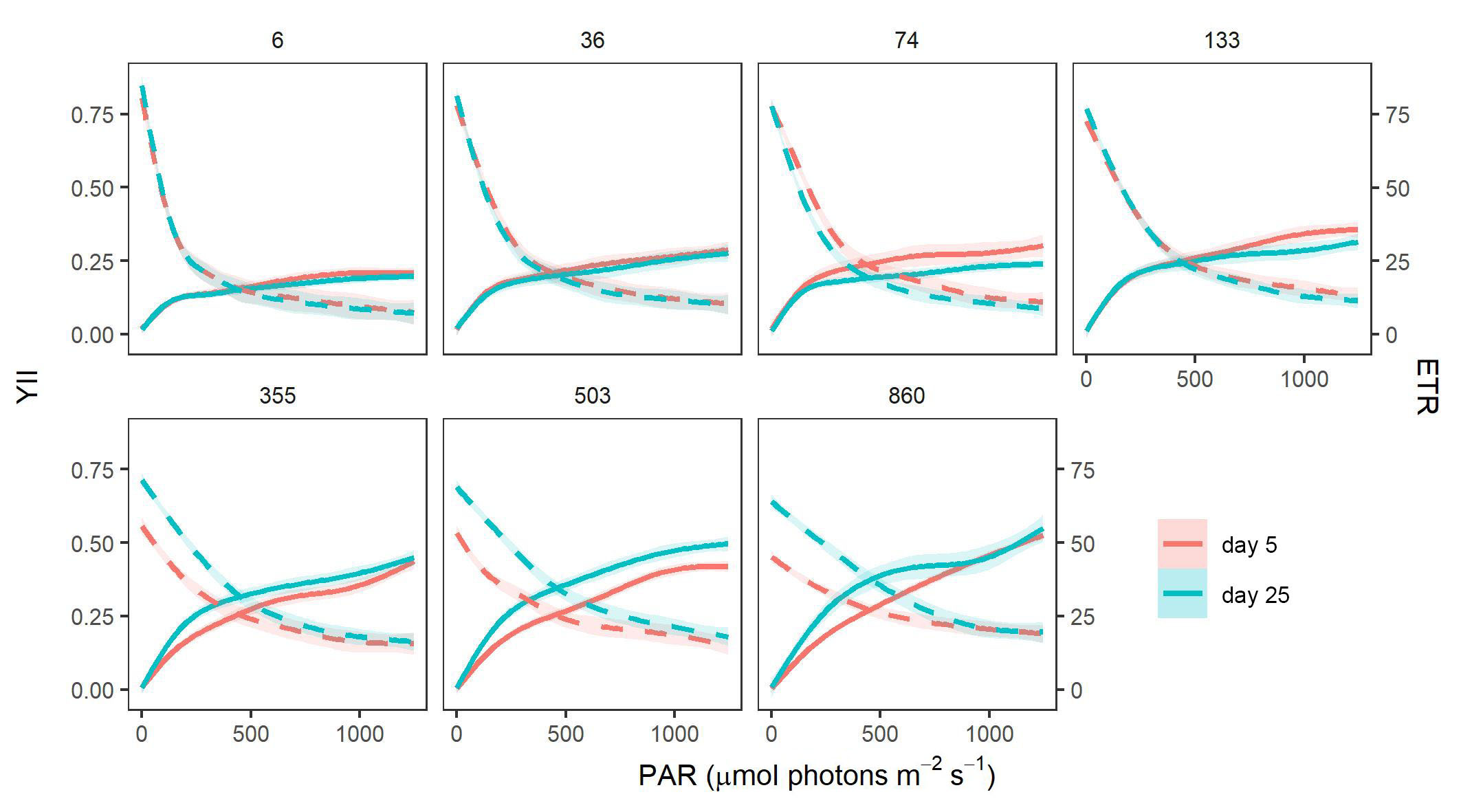

Supplement: Supplementary Figure 2 — Effective quantum yield (YII) of photosystem II (PSII) (dashed lines) and electron transport rate (ETR, solid lines) for each light treatment (μmol photons m–2 s–1) during RLCs at the beginning and the end of the experiment (day 5 in red and day 25 in blue). Lines are mean values (n = 5–9) with a 95% CI. [file Image_2.JPEG]

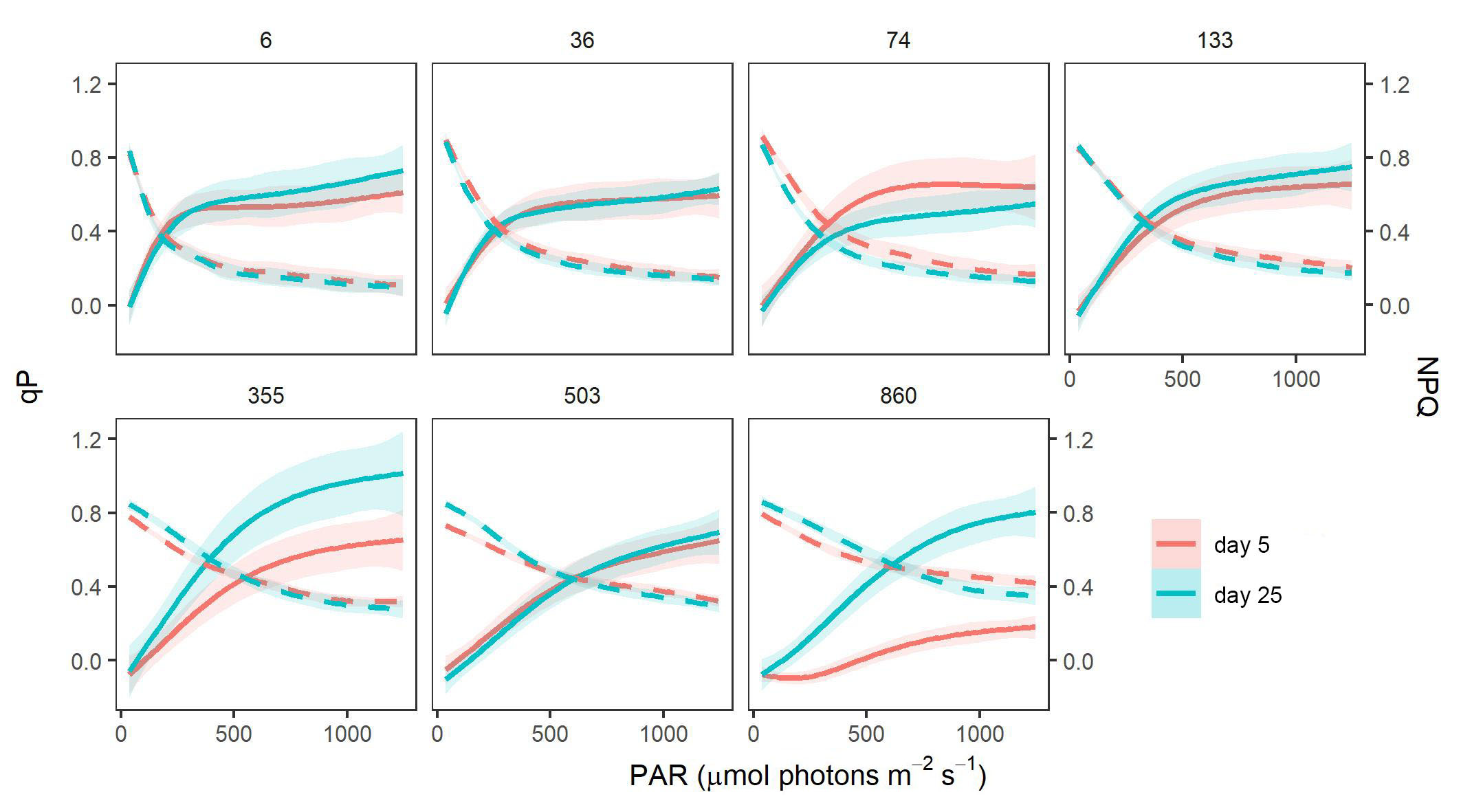

Supplement: Supplementary Figure 3 — Photochemical coefficients (qP, dashed lines) and non-photochemical quenching coefficients (NPQ, solid lines) for each light treatment (μmol photons m–2 s–1) during RLCs at the beginning and the end of the experiment (day 5 in red and day 25 in blue). Lines are mean values (n = 5–9) with a 95% CI. [file Image_3.JPEG]
